# Acupuncture for ophthalmoplegia: Protocol for a systematic review

**DOI:** 10.1097/MD.0000000000011065

**Published:** 2018-06-15

**Authors:** Meiqi Ji, Yali Qin, Yingxin Zi, Rui Wang, Huan Meng, Zongchun Yang, Qi Zhao, Ming Jin

**Affiliations:** a Beijing University of Chinese Medicine; b Department of Ophthalmology, China-Japan Friendship Hospital, Beijing, China.

**Keywords:** acupuncture, ophthalmoplegia, protocol, systematic review

## Abstract

**Background:**

Ophthalmoplegia is a disease that affects many people every year and is caused by reasons, such as cavernous sinus lesion, intracranial aneurysm, diabetes, and trauma. Acupuncture has been widely used to treat ophthalmological diseases especially ophthalmoplegia in China. Many clinical trials indicate that acupuncture may promote the recovery of extraocular muscles in ophthalmoplegia patients. We aim to conduct a meta-analysis to evaluate the efficacy and safety of acupuncture for ophthalmoplegia.

**Methods:**

We will retrieve the literature from the following electronic databases, by March 31, 2018, such as PubMed, EMBASE, the Cochrane Library, Web of Science database, Chinese BioMedical Literature Database, China National Knowledge Infrastructure, China Science and Technology Journal database, and Wanfang Database. We will also collect clinical trial registries, dissertations, grey literature, reference lists of studies, systematic reviews, and conference abstracts. Two people will review these articles, extract the data information, and assess the quality of studies separately. Data will be synthesized by either fixed-effects or random-effects model regarding to a heterogeneity test. The eyeball movement distance, size of fissure palpebrae, and the reduced degree of strabismus will be assessed as the primary outcomes. The secondary outcomes will be the size of the pupil, main symptom scores, ocular localization analysis, and functional impairment extent and safety. We will use the specific software called RevMan (version 5.3) to perform the meta-analysis.

**Results:**

This study will provide a high-quality synthesis based on current evidence of acupuncture for ophthalmoplegia, especially its impacts on eyeball movement distance, size of fissure palpebrae, the reduced degree of strabismus, size of the pupil, main symptom scores, ocular localization analysis, and functional impairment extent and safety.

**Expected conclusion:**

Our systematic review will provide evidence to determine whether acupuncture is an effective and safe intervention for ophthalmoplegia patients.

**Ethics and dissemination:**

It is not necessary for this systematic review to acquire an ethical approval. This review will be disseminated in a peer-reviewed journal or conference presentation.

**PROSPERO registration number:**

PROSPERO CRD42018091536.

## 1 Introduction

Ophthalmoplegia is defined as a complex disease, due to the influence of congenital dysplasia and acquired factors, which usually lead to organic diseases of the eye movement system and the extraocular muscles. It can cause extraocular muscles partially or completely paralysis.^[1]^ Ophthalmoplegia is usually caused by a variety of reasons, due to the third, fourth, and sixth cranial nerve paresis, that may be associated with cavernous sinus lesion,^[2,3]^ intracranial aneurysm,^[4]^ diabetes,^[5]^ Tolosa-Hunt syndrome,^[6]^ migraine,^[7]^ Kearns-Sayre syndrome,^[8]^ myasthenia gravis,^[9]^ trauma,^[10]^ etc. Ophthalmoplegia can be categorized as oculomotor cranial nerve palsy, trochlear cranial nerve palsy, or abducens nerve palsy, all conditions in which the third, fourth, and sixth cranial nerves are affected. The overall prevalence of ophthalmoplegia cases was 0.32% among diabetic patients,^[5]^ along with 0.5% in the Italian studies in 2011,^[11]^ and 0.5% in the Japanese studies. Which are 10 times more frequent than nondiabetic subjects.^[12]^ It should be noted that ophthalmoplegia can also lead to strabismus, diplopia, headache, and ptosis,^[13,14]^ which seriously affect the quality of life and appearance of patients.

Up to now, the treatment for ophthalmoplegia includes medical therapy and operative treatment, such as tetracycline,^[15]^ corticosteroid treatment,^[16]^ botulinum toxin,^[17]^ vitamin,^[18]^ reconstruction of the lateral orbital wall,^[19]^ combination of modest blepharoplasty, and frontalis suspension.^[20]^ Most of the interventions are symptomatic treatments, and the efficacy concerning recovery of ophthalmoplegia has not been assessed adequately.^[4]^ Therefore, there is a demand for more studies to reveal effective treatments for ophthalmoplegia.

As a complementary and alternative therapy, acupuncture has been widely used in various diseases during the past thousands of years in China. According to traditional Chinese Medicine (TCM) theory, acupuncture is a type of treatment, which stimulates acupoints by inserting a filiform needle to regulate the balance of Qi and blood. Previous studies have proven that acupuncture could be applied in a lot of nervous system diseases, such as stroke,^[21]^ vascular dementia,^[22]^ and facial paralysis.^[23]^ In addition, there are many types of research on the treatment of ophthalmoplegia by acupuncture, which has shown that acupuncture Jingming (UB1), Chengqi (ST1), Sibai (ST2), and Qiuhou (EX-HN7) can promote eyeball movement and recover the function of ocular muscles.^[24,25]^

In the preliminary searches of the electronic databases, we found that randomized controlled trials (RCTs) of acupuncture for ophthalmoplegia are on the rise.^[26]^ Because of the limitation of funding and practical clinics, most of the existing literature only includes small sample studies. Therefore, it is necessary to conduct a meta-analysis on ophthalmoplegia to evaluate the efficacy and safety of acupuncture. In this review, we aim to assess the efficacy and safety of acupuncture for treating ophthalmoplegia.

## 2 Methods

This systematic review protocol has been registered on PROSPERO as CRD42018091536 (https://www.crd.york.ac.uk/PROSPERO/display_record.php?RecordID=91536). The protocol follows the *Cochrane Handbook for Systematic Reviews of Interventions* and the Preferred Reporting Items for Systematic Reviews and Meta-Analysis Protocol (PRISMA-P) statement guidelines.^[27]^ We will describe the changes in our full review if needed.

### 2.1 Inclusion criteria for study selection

#### 2.1.1 Types of studies

This review will include clinical RCTs of acupuncture therapy for ophthalmoplegia without any language or publication status restrictions. Non-RCTs, quasi-RCTs, case series, case reports, crossover studies, uncontrolled trials, and laboratory studies will not be included.

#### 2.1.2 Types of participants

People with a diagnosis of ophthalmoplegia will participant without considering any information related to their age, sex, race, education, nationality, or economic status.

#### 2.1.3 Types of interventions

##### 2.1.3.1 Experimental interventions

In the experimental groups, we plan to include the following acupuncture therapies: manual acupuncture, body acupuncture, electroacupuncture, dermal needle, auricular acupuncture, scalp acupuncture, ocular acupuncture, fire needling, warm needling, and plum blossom needle. Pharmacoacupuncture, acupoint injection, laser acupuncture, moxibustion, cupping, and transcutaneous electrical nerve stimulation will be excluded. In addition, there is no limitation to the test cycle and treatment frequency.

##### 2.1.3.2 Control interventions

In the control groups, we plan to use the categories: no treatment, sham acupuncture, placebo acupuncture, and drug therapy. We will include studies that have compared acupuncture plus another therapy with the same other therapy alone. We will also exclude RCT which have compared 2 different types of acupuncture or compared acupuncture with TCM, moxibustion, and other TCM treatment.

The following treatment comparisons will be addressed:

1. Acupuncture versus no treatment.
2. Acupuncture versus placebo/sham acupuncture.
3. Acupuncture versus drug therapy.
4. Acupuncture versus other therapies.
5. Acupuncture plus another therapy versus the same other therapy alone.

#### 2.1.4 Types of outcome measures

##### 2.1.4.1 Primary outcomes

The primary outcome measurement will be an improvement of eyeball moving distance, size of fissure palpebrae, and the reduced degree of strabismus. The eyeball moving distance and size of fissure palpebrae will be measured by a ruler. The ruler unit is accurate to the millimeter. The reduced degree of strabismus is evaluated by a validated scale, such as Strabismus scale.

##### 2.1.4.2 Secondary outcomes

The secondary outcomes of the review will include as followed:

1. Size of the pupil.
2. Main symptom scores before and after treatment.
3. Ocular localization analysis and functional impairment extent.
4. Safety: measured by incidence and severity of side effects.

### 2.2 Search methods for the identification of studies

#### 2.2.1 Electronic searches

We will retrieve the literature from the following electronic databases, by March 31, 2018, such as PubMed, EMBASE, the Cochrane Library, Web of Science database, Chinese BioMedical Literature Database, China National Knowledge Infrastructure, China Science and Technology Journal database, and Wanfang Database. According to the *Cochrane Handbook Guidelines*, all the reviewers will discuss the search terms and search strategies. The following search terms will be used: acupuncture, acupuncture therapy, acupoints, manual acupuncture, body acupuncture, electro-acupuncture, electroacupuncture, dermal needle, skin acupuncture, ear acupuncture, auricular acupuncture, scalp acupuncture, ocular acupuncture, fire needling, warm needling, plum blossom needle, ophthalmoplegia, ophthalmoparesis, oculomotor paralysis, abducens paralysis, trochlear palsy, external ophthalmoplegia, and diplopia. The equivalent search terms will be translated into Chinese while searching in the Chinese databases. The search strategy for PubMed is shown in Table 1.

**Table 1:**
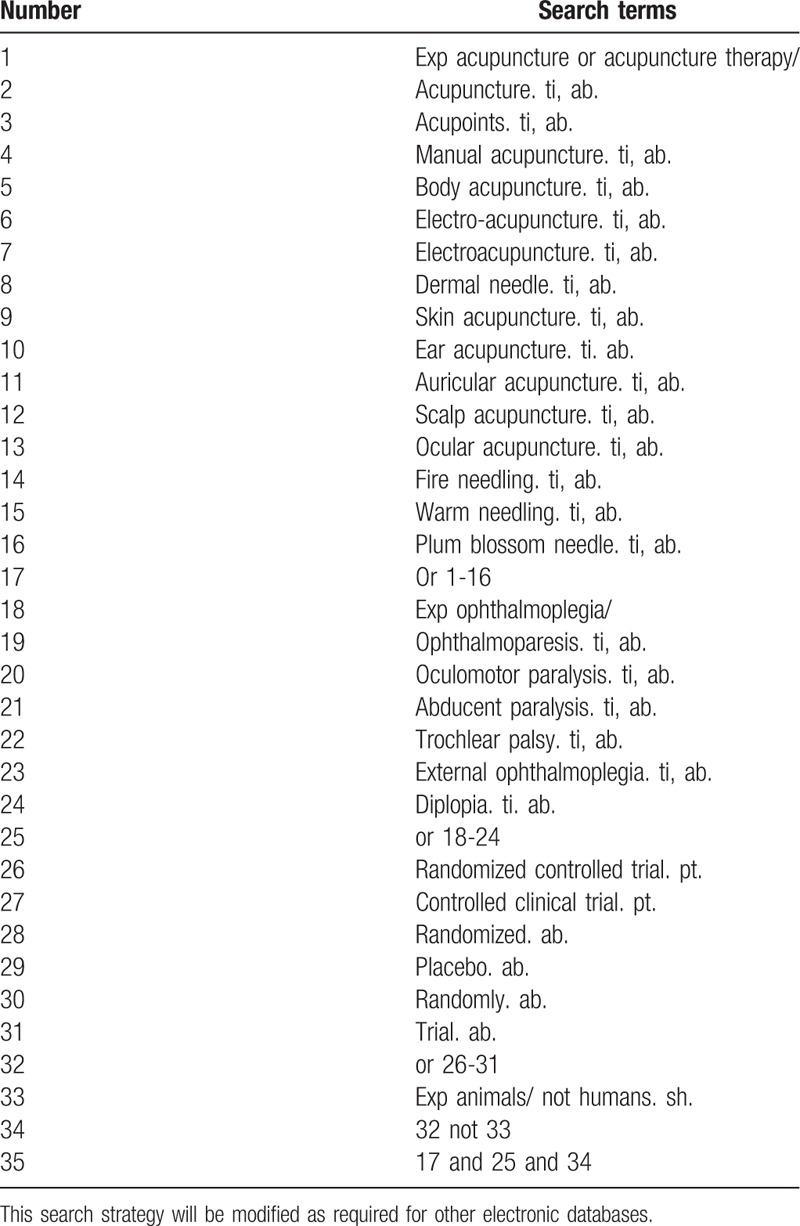
Search strategy used in PubMed database.

#### 2.2.2 Searching other resources

In addition, the clinical trial registries, dissertations, and grey literature will be searched. For additional trials, we will search the reference lists of studies, systematic reviews, and conference abstracts related to acupuncture and ophthalmoplegia. Ongoing trials for the new reviews which relevant to this term will be retrieved from the World Health Organization International Clinical Trials Registry Platform (ICTRP), ClinicalTrials.gov, and the Chinese Clinical Trial Registry. We will also search in OpenGrey.eu. Web site for potential gray literature.

### 2.3 Data collection and analysis

#### 2.3.1 Selection of studies

**Figure 1:**
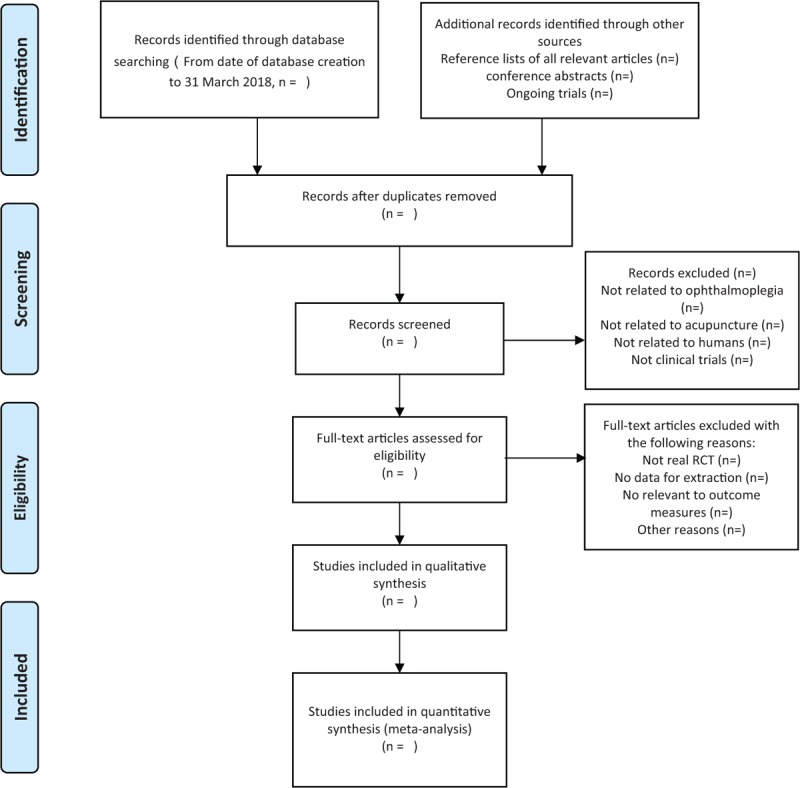
The PRISMA flow chart. RCT = randomized controlled trial. *From:* Moher D, Liberati A, Tetzlaff J, Altman DG, The PRISMA Group (2009). *P*referred *R*eporting *I*tems for *S*ystematic Reviews and *M*eta-*A*nalyses: The PRISMA Statement. PLoS Med 6(6): e1000097. doi:10.1371/journal.pmed1000097.

Before selection of studies, all reviewers must get trained to understand the purpose and process of the review. We will use EndNote X8 software to manage the records, which searched electronic databases and other resources. Two reviewers (MQJ and ZCY) will screen the titles, abstracts, and keywords of all retrieved records separately. Full texts will be examined if necessary based on the inclusion criteria. All reviewers will screen the name of the author, institution, and journal of publication. The excluded studies will be listed by a named table “reasons for excluded studies.” Any disagreements should be resolved through discussion to get a consensus and judged by an arbiter (MJ). The study selection procedure is shown in the PRISMA flow chart (Fig. 1).

#### 2.3.2 Data extraction and management

According to the inclusion, a standard data collection form will be made before data extraction. Two reviewers (MQJ and ZCY) will independently extract data from the selected studies and fill in the data collection form. The discrepancies and uncertainties will be resolved to get a consensus by consulting a senior reviewer (MJ) through discussion. We will contact the corresponding authors for more information if the details of the trials were not completed. All data will be cross-checked by MQJ and ZCY and transferred into review manager software. The following data will be extracted:

1. General information: research identification, publication year, the title of the study, first author, correspondent author, contact information, the name of the journal, country.
2. Study methods: study design, sample size, randomization method, allocation concealment, blinding, incomplete report or selecting report, other sources of bias.
3. Participants: inclusion and exclusion criteria, age, sex, race, severity, research site, the baseline of ophthalmoplegia, diagnostic criteria of ophthalmoplegia.
4. Interventions: type of acupuncture/control, needles, acupoints, chemotherapy drugs, other treatment, treatment details, treatment duration, and frequency.
5. Outcomes: primary, secondary, and safety outcomes as described above.
6. Notes: financial support, conflicts interest, ethical approval, important citations.

#### 2.3.3 Assessment of risk of bias in included studies

The risk and bias in included studies will be assessed independently by 2 reviewers (YLQ and YXZ) using the risk of bias (ROB) assessment tool in the *Cochrane Handbook*. The following domains will be assessed: random sequence generation, allocation concealment, blinding of participants and personnel, blinding of outcome assessment, incomplete outcome data, selective outcome reporting, and other bias. We will give each index a low bias, unclear, or high bias, and any discrepancies will be resolved by a discussion between the 2 reviewers or consulting a third reviewer (QZ).

#### 2.3.4 Measures of treatment effect

For continuous data, we will use mean difference (MD) or standardized mean difference with 95% confidence interval (CI). For dichotomous outcomes, we will use the relative risk (RR) and 95% CI records.

#### 2.3.5 Unit of analysis issue

We will assess the first experimental period data in cross-over trials to prevent carry-over effects. With multiple intervention groups, we will combine all relevant experimental and control intervention groups into a single respectively to avoid a unit of analysis issue.

#### 2.3.6 Dealing with missing data

If data information is missing in the included studies, 2 reviewers (RW and HM) will contact the first authors to obtain the missing data. If the missing data are unobtainable, we will analyze the available data and describe it in the discussion. We will discuss the impact of missing data if necessary.

#### 2.3.7 Assessment of heterogeneity

We will use complete case data as analysis data. Heterogeneity will be assessed by a standard χ^2^ texts with a significance level of *P* < .1 and *I*^2^ test. When the *I*^2^ value is <50%, the study will be considered to have no statistical heterogeneity, and the fixed-effect model will be selected. Although *I*^2^ ≥50%, the study will be considered to have substantial heterogeneity, and we will select a random-effect model.

#### 2.3.8 Data synthesis and analysis

We will use Review Manager software (RevMan V.5.3.5) provided by Cochrane Collaboration for data synthesis and analysis. When *I*^2^ <50%, a fixed-effects model will be used to calculate the RR and MD. When *I*^2^ ≥50%, we will use a random-effects model to synthesize the data. Subgroup analysis will be performed and the potential reasons will be analyzed to explore the causes of heterogeneity. If meta-analysis is not appropriate, we may use narrative synthesis.

#### 2.3.9 Assessment of publication bias

When 10 or more studies are in the meta-analysis, we will use funnel plots and statistic test to detect publication bias.

#### 2.3.10 Subgroup analysis

If we identify substantial heterogeneity, we will perform subgroup analysis for different intervention forms. We will take acupuncture therapy types, the degree of ophthalmoplegia severity, the age of patients, and other different control interventions into consideration.

#### 2.3.11 Sensitivity analysis

We will perform sensitivity analysis for primary outcomes to test the robustness of the review conclusions, and we will still evaluate the impact of methodological quality, sample size, and missing data.

#### 2.3.12 Grading the quality of evidence

We will use the Grading of Recommendations Assessment, Development, and Evaluation to evaluate the quality of confidence for primary outcomes in including studies.^[28]^ The quality of evidence will be classified into “very low,” “low,” “moderate,” or “high” judgment.

## 3 Discussion

Ophthalmoplegia is a complex disease in ophthalmology and neurology. Nowadays, drug and surgical treatment are the most commonly used treatments for ophthalmoplegia. However, some patients with ophthalmoplegia would like to find nonpharmacological interventions with low side effects to improve the symptoms.

With its minimal side effects and low financial cost, acupuncture has been used to treat ophthalmological diseases for many years in China, such as hordeolum,^[29]^ optic atrophy,^[30]^ and oculomotor palsy. As a complementary and alternative treatment based on TCM, acupuncture stimulates the acupoints to move the vital Qi through the meridians of the body and also regulate the balance of Yin and Yang.^[31]^ However, the mechanisms of acupuncture are still unclear in treating ophthalmoplegia. Despite this lack of knowledge of a clear mechanism, results of clinical studies suggest that acupuncture could recover the function of eyeball movement and visual sense.^[32]^

Nevertheless, there has been no complete evaluation of the clinical evidence concerning acupuncture interventions for ophthalmoplegia based on evidence-based medicine. Therefore, we intend to conduct this systematic review and meta-analysis to evaluate the efficacy and safety of acupuncture treatment for ophthalmoplegia patients. We hope that this systematic review will provide more evidence to help patients and clinicians to make right choices when dealing with ophthalmoplegia.

It should be noted that there might be limitations in this review. First, the use of language including English and Chinese may induce the bias of the study. Second, different types of acupuncture, acupoints, duration, frequency, the age of patients, and degree of ophthalmoplegia severity may cause high heterogeneity. Third, it is difficult to undertake single or double-blind experiment measures during acupuncture therapy.

## Author contributions

**Conceptualization:** Ming Jin.

**Data curation:** Meiqi Ji, Yali Qin, Zongchun Yang, Qi Zhao.

**Resources:** Yingxin Zi, Rui Wang, Huan Meng.
